# Dissemination of a computer-based psychological treatment in a drug and alcohol clinical service: an observational study

**DOI:** 10.1186/1940-0640-9-15

**Published:** 2014-08-09

**Authors:** Frances J Kay-Lambkin, Aaron L Simpson, Jenny Bowman, Steven Childs

**Affiliations:** 1 National Drug and Alcohol Research Centre, University of New South Wales, Sydney, Australia; 2 Centre for Translational Neuroscience and Mental Health, University of Newcastle, Newcastle, Australia; 3 School of Psychology, University of Newcastle, Newcastle, Australia; 4 Central Coast Drug and Alcohol Clinical Service, Northern Sydney Central Coast Area Health Service, Gosford, Australia

**Keywords:** Computer/internet-based psychological treatment, Computer anxiety, Openness to innovation, Alcohol/other drug clinical service

## Abstract

**Background:**

There is emerging evidence for the potential of computer-based psychological treatments (CBPT) as an add-on to usual clinical practice in the management of health problems.

**Objective:**

The study set out to observe if, when, and how clinicians working in a publically funded alcohol/other drug (AOD) clinical service might utilize SHADE (*S*elf-*H*elp for *A*lcohol and other drug use and *DE*pression), a CBPT program for comorbid depression and alcohol or cannabis use, in their clinical practice.

**Methods:**

Thirteen clinicians working within an AOD service on the Central Coast of New South Wales, Australia, were recruited. At baseline, all 13 clinicians were assessed for their computer anxiety and openness to innovation. Clinicians referred current clients to the study, with consenting and eligible clients (N = 35) completing a baseline and 15-week follow-up clinical assessment. The assessment comprised a range of mental health and AOD measures administered by an independent research assistant. Over the course of the study, clinicians submitted session checklists detailing information about session content, including the context and extent to which SHADE was used for each client.

**Results:**

Descriptive statistics showed that clinicians employed the SHADE program in a variety of ways. When SHADE modules were used, they were generally introduced in the early phase of treatment, on average, around session 4 (M = 3.77, SD = 5.26, range 1–36). However, only 12 of the 35 clients whose session checklists were available were exposed to the SHADE modules; this, despite 28/35 clients indicating that they would be willing to use CBPT during their current treatment program.

**Conclusions:**

Treatment seekers in the AOD service of the current trial were generally open to receiving CBPT like SHADE; however, clinicians tended to use SHADE with only 34 percent of clients. This indicates the importance of providing ongoing support and encouragement to clinicians, in addition to an initial training session, to encourage the adoption of innovative technologies into clinical practice, and perhaps to engage clients in a discussion about CBPT more routinely.

**Trial registration:**

Australian Clinical Trial Registration Number ACTRN12611000382976.

## Background

With the rise and global impact of mental health and alcohol/other drug (AOD) use disorders, new avenues for treatment have revealed the potential for technology to respond to this increasing need. For example, a recent meta-analysis of the use of internet/computerized interventions for problem drinking [1] identified nine randomized controlled trials (RCTs) attesting to the efficacy of these treatment modalities in reducing alcohol use. Similarly, a meta-analysis of computerized treatments for depression and anxiety disorders [2] identified 22 RCTs demonstrating that computer therapy for these disorders is effective and acceptable.

There are many potential benefits of incorporating computer-based psychological treatments (CBPT) into community-based mental health services (including drug and alcohol clinical services). The standardized nature of automated treatment can improve the transportability of evidence-based practice from research to real-world clinical environments, and it lowers many of the barriers people face to accessing care. The technology also offers a level of convenience not generally available via therapist-delivered treatment (such as 24-hour access to treatment, no wait-lists). Within a stepped-care framework, CBPT offers an alternate entry point into mainstream treatment or a credible alternative for people who cannot or choose not to seek treatment from existing mental health services [3,4].

The potential benefits of CBPT for clinicians and the broader health care system are equally significant. Demand for psychological treatment outstrips supply, particularly in regional and rural communities. By delegating parts of treatment to a computer, clinicians may be able to redirect their time and expertise toward those patients who require more intensive and/or specialist intervention [4]. Marks and colleagues [5] compared treatment outcomes for people with panic disorder receiving computer-delivered, therapist-delivered, or combined therapist/computer-delivered treatment. By delegating self-exposure tasks to a computer, clinicians reduced the amount of face-to-face time per client by 73 percent, without compromising treatment outcomes [5].

Kay-Lambkin and colleagues [6] developed and evaluated a CBPT package for co-existing depression and alcohol/drug use disorders: SHADE (*S*elf-*H*elp for *A*lcohol and other drug use and *DE*pression). After completing a face-to-face assessment comprising feedback, case formulation, and initial goal setting, participants (n = 97) were randomly allocated to either: 1) therapist-delivered cognitive behavioral therapy (CBT) and motivational interviewing (MI); 2) integrated computer-delivered CBT/MI (i.e., SHADE plus brief weekly therapist assistance); or 3) one session of feedback and case formulation delivered face to face as the control condition (brief intervention). The study found no significant differences between the intensive therapist-delivered CBT/MI and that delivered primarily via the SHADE computer program, with significant improvements in depression, drug use, and quality-of-life measures for both conditions at 12-month follow-up. Both CBT/MI conditions (therapist- and computer-delivered) were associated with greater (nonsignificant) reductions in depression, alcohol, and cannabis use relative to the brief intervention condition, with computer-delivered SHADE treatment associated with greater (nonsignificant) reductions in cannabis use over the other treatment conditions. Computer-delivered SHADE treatment required on average 12.5 minutes of generic face-to-face time per session, compared to 60 minutes of specialist psychologist input for the therapist-delivered CBT/MI treatment. A study examining client acceptability of the SHADE program found that people receiving CBPT are equally able to engage, bond, and commit to treatment as those receiving therapist-delivered treatment [7].

In a replication of the initial SHADE study [8], 274 participants were recruited across seven urban and rural communities, but using a different active control condition of 10 sessions of supportive counseling (face to face, without any CBT/MI strategies) rather than the brief intervention control. Like the initial study, computer- and intensive therapist-delivered CBT/MI were associated with superior reductions in depression, alcohol, and cannabis use compared to supportive counseling, indicating that improvements were not simply the product of nonspecific effects. This research promises much for the practical application of CBPT, particularly the SHADE program, as an additional strategy in the treatment of co-existing depression and drug use problems. CBPT has the potential to be a ‘clinician extender’ and sit credibly along a continuum of evidence-based psychological care [5].

Despite the potential and emerging evidence for CBPT, its uptake into clinical practice has been low [9,10]. This may be due to the scarcity of research into the barriers, expectations, and experiences of both clinicians and clients when using CBPT in a clinical setting [11].

As with any treatment, there will be a range of client- and clinician-level factors influencing the uptake, use, and impact of CBPT. It remains unclear if the technology will realize its potential. Our study set out to complete an observational field study of CBPT use, namely the SHADE resource, in a community-based drug and alcohol clinical service. Our primary aim was to observe if, when, and how clinicians might utilize SHADE in their clinical practice, following provision of a 2-hour information and training session on the SHADE program. We aimed to understand the relationship between use of the SHADE program and clinician attitudes towards technology. We also set out to explore changes in key outcomes for clients exposed to the SHADE resource, along with self-reported willingness of clients to use CBPT in their treatment.

## Methods

### Setting and design

The study was undertaken in collaboration with the Central Coast Alcohol and Other Drug Service (CCAODS), a subsidiary of the Northern Sydney Central Coast Area Health Service. CCAODS delivers a wide range of AOD treatment services, including inpatient and community detoxification, pharmacotherapy programs, general counseling, medical services, a court-mandated diversion program for people with drug use problems and concurrent legal issues (Magistrates Early Referral Into Treatment; MERIT), as well as a specialist program for clients with a primary drug concern of marijuana misuse/dependency (Cannabis Clinic). The service also provides general practitioner liaison, health promotion, community consultation, and an Aboriginal liaison services. A central intake service acts as the point of initial contact with the CCAODS, with referrals being forwarded to relevant departments. The type of psychological intervention provided by clinical staff is not prescribed. All clients entering the service complete a standardized intake assessment. All CCAODS clinicians attend regular review sessions and compulsory clinical supervision.

Eight desktop computers were supplied to the CCAODS for the duration of the study. Each computer had the SHADE–CBPT program pre-installed onto its hard drive. These computers were not connected to the internet. No conditionality or instructions were supplied with the computers, and clinicians were free to make use of the computers however they wished. Most counseling rooms also had an internet-compatible desktop computer. An unlimited supply of SHADE DVDs was also made available throughout the study for clients to take home and complete at the discretion of the clinician. Clients did not have to participate in the research to use SHADE.

The study was employed as an observational study of clinician implementation of SHADE with current and ongoing clients in their caseload, following a 2-hour information and training session on the resource.

#### SHADE – computer-based psychological treatment

The SHADE–CBPT program has been described elsewhere [6,8]. SHADE incorporates CBT/MI strategies to encourage reductions in depression and AOD use. SHADE is available in two formats: 1) a 10-session program designed to be completed in a linear fashion, with content pre-programmed for each session; or 2) a skill module program, where a series of shorter modules are presented based on themes related to depression and AOD use problems (e.g., coping with cravings, taking charge of one’s thoughts, staying well) arising from the 10-week program. Clients or clinicians are able to select a particular skill module to focus on during a session, without having to complete the other skills and strategies contained in the resource. Both versions of SHADE were made available for the study. Text is pitched at a reading age of 14 years, with a voiceover available to read out all text contained in the resource. Video case scenarios guide clients through a range of CBT/MI skills and strategies, and accompanying handouts and worksheets are also available for clients/clinicians to print out and use during a session or as a homework exercise.

### Participants

#### Clinicians

All clinicians (n = 13) working within the CCAODS – Drug and Alcohol Counseling, Cannabis Clinic, and MERIT teams were invited to participate in the study. All clinicians had tertiary qualifications in a counseling-related field, with at least an undergraduate degree in nursing or psychology. The group reported a mean age of 42.90 years (SD = 11.17, range 25–58) and were, for the most part, female (n = 11/13). Clinicians provided assessment and treatment according to evidence-based psychosocial guidelines established for their service [12].

#### Clients

All new and existing adult clients seeking treatment from CCAODS’ Drug and Alcohol Counseling, Cannabis Clinic, and MERIT services were recruited for a larger study of treatment outcomes in the service, with separate consent and assessment procedures provided through their clinician [13]. The study team was responsible for recruiting clients into the study, based on a referral from their treating clinician to the research team. To preserve anonymity and the confidentiality of participants and assessment results, clinicians were not aware of the participation status of their clients in the trial; in addition, clinicians were asked to submit session checklists for *all* clients throughout the study period.

### Measures

The assessment tools used as part of the study are widely used in mental health and/or AOD treatment research and practice. A full description of the study methods has been published previously [13]. Ethics approval for the study was obtained from Northern Sydney Central Coast Human Research Ethics Committee (08/HARBR/78/79), the University of Newcastle Human Research Ethics Committee (H-2008-0271), and the Macquarie University Ethics Review Committee (Human Research, 0806-125 M(R)).

#### Clinician measures

The following measures were completed at baseline only, prior to receipt of any CBPT material:

Individual Opinion Scale IOS [14]; – a 20-item measure using a 7-point Likert scale assessing the likelihood of an individual to adopt innovative strategies in their work. The more innovative an individual believes they are, the higher their score on the IOS. Scores on the IOS range from 20 to 140. The IOS has a reliability of 0.94, with acceptable construct and predictive validity. The measure has an internal consistency reliability of 0.88.

Computer Opinion Survey (CAIN) – a measure of computer anxiety and a proxy criterion for how comfortable clinicians are with adopting and using CBPT. The CAIN is a 26-item measure using a 6-point Likert scale, with scores ranging from 26 (the highest level of computer anxiety) to 156 (measure of least computer anxiety). Internal consistency of the scale is 0.94, with test/retest reliability of 0.90 [15].

#### Client measures

Demographic details (e.g., age, gender, employment status, mental health treatment history) were recorded at baseline. Client assessment collected at baseline and at 15-week follow-up included:

Opiate Treatment Index OTI [16]; – The OTI assesses the quantity and frequency of use for 11 different drugs, including: alcohol, cannabis, heroin, other opiates, amphetamines, cocaine, hallucinogens, barbiturates, tranquilizers, inhalants, and tobacco. Each of the 11 drug types are assessed individually, and clients report on their last three using occasions in the month prior to assessment, estimating the amount of drug consumed on each of these occasions. An average use index for the previous month is calculated for each drug. Use of alcohol and cannabis, the most frequently reported drugs used by clients of the service, are reported here.

Depression Anxiety Stress Scale, 21-item version DASS-21 [17]; – The DASS-21 was used to measure depression, anxiety, and stress scores for the 2 weeks prior to assessment. The scale has a Cronbach alpha of 0.93 for the total measure, as well as high reliability for the subscales of stress, depression, and anxiety (0.93, 0.90, and 0.82, respectively) [18].

Previous experience of computers/the internet for AOD treatment and openness to integrating technology into current treatment plan – At baseline, clients were asked a series of questions about their previous computer experience and their willingness to try computerized treatments in their current AOD treatment program.

### Procedures

#### Clinicians

Clinicians attached to each of the counseling teams participating in the study attended an information session facilitated by the authors. Clinicians were provided with an overview of the study, a brief appraisal of research supporting CBPT as a treatment option for mental health problems (including AOD use problems), and a demonstration of the SHADE treatment program as an example. The group discussed different ways in which SHADE could be incorporated into the clinical practice, either using the full 10-session program or individual SHADE sessions (e.g., on relapse prevention, mindfulness, or problemsolving) to supplement the ongoing treatment plan. This session lasted for 2 hours.

Throughout the study, participating clinicians completed four key activities:

1. At baseline, and prior to receiving the CBPT training session, clinicians completed two self-report measures assessing their openness to innovation and computer anxiety.

2. Following baseline assessment and receiving the CBPT training session, clinicians were asked to consider and use SHADE with new and ongoing clients in whatever manner they chose.

3. Following baseline assessment and receiving the CBPT training session, clinicians were asked to forward the contact details for new and ongoing clients to the research team throughout the study period, regardless of their exposure to SHADE.

4. At the conclusion of each session during the study period, the clinicians were asked to complete a session checklist. The checklist collected information about the focus and content of the session, including whether or not SHADE was discussed, used in-session, and/or recommended as a homework exercise. The checklist was developed by the authors to specifically suit the CCAODS and the range of counseling interventions used by the clinicians.

#### Clients

The recruitment and consent process for clients was conducted by the research team, independently from the clinicians participating in the study. Following referral from the clinicians in the CCAODS, the research team contacted potential clients to discuss study participation and obtain consent. Study participation involved completing a baseline and a 15-week follow-up assessment delivered over the telephone by a research assistant, independent from the CCAODS. Clients were reimbursed $20AUD for each completed assessment. For the current study, eligible clients were those who provided consent to participate in the baseline and 15-week follow-up assessments and who had at least one clinician checklist submitted to the team for analysis.

### Data analysis

Data was analysed using SPSS, version 17.0 (SPSS Inc., Chicago, IL, USA).

#### Use of SHADE

Descriptive statistics explored the self-reported use of SHADE by clinicians involved in the project as a function of primary drug of concern, and where and how it was used in the treatment protocol.

#### Clinician innovation and computer anxiety

Descriptive statistics were used to explore the responses of clinicians to these surveys, with one sampled t-test employed to examine potential differences between score on these scales in the sample, relative to the established norms for each scale. Pearson correlation coefficients were calculated to examine the relationship between scores on the IOS and those on the CAIN.

#### Patterns of change in key client outcomes

Descriptive statistics summarized client responses to prior computer use and their openness to receiving computerized treatments in the current treatment program, along with patterns of change in alcohol use, cannabis use, depression, anxiety, and stress scores over the study period.

## Results

### Client and clinician participants

**Figure 1:**
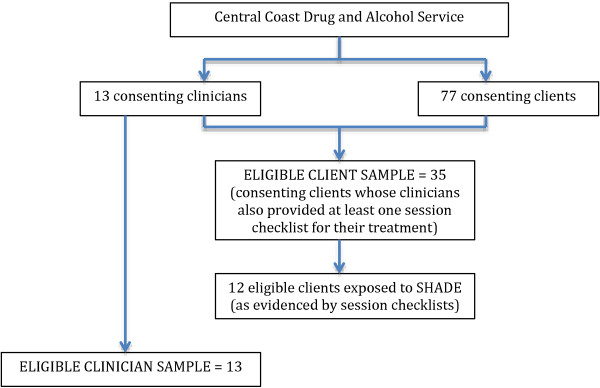
The number of clinicians and clients of a publicly funded drug and alcohol service who provided data for the current study.

Figure 1 displays the number of clinicians and clients providing data for the study.

For the current analysis, eligible clients were those for whom their treating clinician had submitted at least one clinician checklist summarizing the session content (N = 35). This represented 45 percent (35/77) of the clients recruited to the larger study. The pool of eligible clients (N = 35) reported a mean age of 42.11 years (SD = 12.152, range 19–65) and included more males than females (n = 22, 63% male).

Of the eligible client pool (N = 35), a review of session checklists revealed that 12 were exposed to the SHADE program during their treatment period.

### Use of CBPT

Clinicians submitted session checklists for 35 clients over the course of the study. Twelve of the 13 clinicians (92%) reported use of the SHADE materials with a client. Clinicians reported using the SHADE program in one of three ways:

1) Introducing and discussing the SHADE resource with clients and recommending its use as part of the client’s treatment plan (n = 3 clinicians);

2) Using the SHADE handouts and worksheets either as homework or to supplement work done within the session (n = 12 clinicians); or

3) Use of the full SHADE modules in-session to deliver a specific psychological treatment/intervention (n = 11 clinicians). In this context, full SHADE modules were used during the session, assigned as homework, or as a combined in-session strategy and homework. Twelve clients (12/35, 34%) were exposed to the full SHADE modules.

If full SHADE modules were used, they were generally introduced in the early phase of treatment; on average, at session 4 (M = 3.77, SD = 5.26, range 1–36). Of the exposed clients (n = 12), an average of 5 of the 10 SHADE modules were completed. If SHADE was discussed rather than the full module being incorporated into sessions, this tended to be later in the treatment experience, and on average was reported during sessions 5–6 (M = 5.26, SD = 8.39, range 1–33).

### Clinician openness to innovation

Clinicians in the study reported an average innovation score on the IOS of 97.62 (SD = 14.32, range 72–130). On average, this was lower than the established norm for the scale of 102, but this difference was not statistically significant (t(12) = −1.104, p = 0.291). Mean IOS scores were 97.92 (SD = 14.95) for 12 clinicians utilizing SHADE in their sessions with clients, with the one clinician choosing not to use SHADE and scoring 94.00 on this scale.

### Clinician trait computer anxiety

Clinicians were relatively low on computer-related anxiety, scoring an average of 120.38 on the CAIN (SD = 17.65, range 91–148). The group rated themselves significantly lower on computer anxiety than did those in the normative sample for the scale (120.38 vs. 105.4, t (12) = 3.046, p = 0.010). The clinician not using SHADE in sessions reported a CAIN score of 108.00, with the 12 clinicians utilizing SHADE reporting a mean of 121.33 (SD = 18.02), with higher scores indicating lower levels of anxiety.

There was not a significant relationship found between scores on the CAIN and scores on the IOS for the sample, indicating no relationship between clinician innovation and computer anxiety (r = 0.455, p = 0.188).

### Willingness to use CBPT

Of the 35 eligible clients for the current study, 31 percent (n = 11) reported previously using computers or the internet to search for information and treatment for a mental health or AOD use problem. Thirty-three clients (94%) felt that integrating a computer-based program into their current treatment plan would be “*a little”* through to *“very”* helpful in treating their current AOD use problems. The majority of clients (n = 28/35, 80%) felt that if they were offered access to a computer-based treatment program during their current treatment plan, they would definitely utilize it, leaving seven clients (20%) not prepared to consider using this form of treatment. For these seven clients, four reported they *“didn’t like technology”* or were *“computer illiterate,”* with the remaining three preferring human contact over that with a computer program.

Of the 35 eligible clients, 12 were exposed to the full SHADE modules, five were exposed to just the SHADE handouts or worksheets rather than a full module, and three has the SHADE resources mentioned to them during a session. Twelve (34%) would have used CBPT, but were not exposed, and three (9%) were unwilling to use CBPT but were exposed to the SHADE handouts or worksheets.

### Patterns of change in key client outcomes

For alcohol use between baseline and 15-week follow-up assessment, participants who did not receive the SHADE modules (n = 23) reported a 3-standard-drink per day reduction in alcohol use between baseline and at 15-week follow-up assessment, while those who were exposed to SHADE (n = 12) reported an 8-standard-drink per day reduction over the same time period.

Clients reported reductions in cannabis use between baseline and 15-week follow-up. For those exposed to the SHADE resource during treatment (n = 12), this was a reported 9-standard-use per day reduction in cannabis use over time, relative to a 3-standard-use per day reduction in those daily users not exposed to SHADE (n = 23).

Table 1 displays the changes in depression, anxiety, and stress scores measured by the DASS-21 over time as a function of use of the SHADE material, along with the changes in alcohol and cannabis use for all eligible participants (N = 35).

**Table 1:** Changes in key client outcomes between baseline and 15-week follow-up assessments according to SHADE exposure (yes/no)

	**Baseline**	**15-weeks post-baseline**
**Domain**	**Mean (Standard Dev.)**	**Mean (Standard Dev.)**
**Alcohol use**		
No exposure to SHADE (n = 23)	6.58 (8.30)	3.90 (4.15)
Exposure to SHADE (n = 12)	11.81 (20.17)	3.76 (4.27)
**Cannabis use**		
No exposure to SHADE (n = 23)	11.15 (9.77)	7.93 (9.17)
Exposure to SHADE (n = 12)	18.63 (16.84)	9.94 (6.33)
Depression*		
No exposure to SHADE (n = 23)	20.69 (2.71)	11.09 (2.11)
Exposure to SHADE (n = 12)	19.83 (3.76)	11.17 (2.93)
Anxiety*		
No exposure to SHADE (n = 23)	11.48 (2.50)	8.44 (2.04)
Exposure to SHADE (n = 12)	14.17 (3.46)	8.83 (2.83)
Stress*		
No exposure to SHADE (n = 23)	22.35 (2.13)	14.17 (2.00)
Exposure to SHADE (n = 12)	24.00 (2.95)	14.50 (2.76)

*Subscale scores on the Depression Anxiety Stress Scale 21 [18].

## Discussion

Improving access to evidence-based treatments for mental health disorders, including AOD problems, is a significant health care priority. CBPT offers potential to address this need. Our study set out to observe how AOD clinicians used CBPT in a real-world clinical setting.

### Utilization of SHADE

Over the course of the study, 12 of the 13 participating clinicians introduced SHADE to their clients, in some form or another. This equated to 12 unique clients (of the 77 consenting to participate in the larger study) who were exposed to at least one full SHADE module (average of 5 of the 10 SHADE sessions). CBPT was commonly introduced to clients early in treatment, most commonly during session 4. This pattern of use may indicate the need for clinicians to establish rapport with their clients prior to suggesting CBPT, and perhaps for immediate crisis-related issues to be addressed first. Some clinicians did introduce SHADE for clients already engaged in treatment (i.e., 10 sessions attended or more); however, most often it was via the use of handouts or worksheets produced by the SHADE program, rather than delegating parts of treatment to CBPT. Our results echo an earlier study indicating that, in the absence of incentives or encouragement from researchers, AOD clinicians tend to refer only a small proportion of their caseload to CBPT [11]. During the 2-hour training and information session provided to clinicians on the SHADE resource, no direct or specific advice was provided as to when or how clinicians should use the program with clients. Clinicians were asked to consider using SHADE with all of their clients, at their discretion, and were provided with scenarios during the training that included using the full 10-session version of the program, using one or two individual modules of the program to supplement their existing treatment plan. It is interesting that each clinician seems to have used SHADE with only one client from their caseload (e.g., 12 clinicians reporting use of SHADE, 12 clients exposed to SHADE). It is possible that clinicians operationalized their commitment to the study as using the SHADE resource with at least one client, rather than this being the minimum. In the context of a busy clinical service, this is not surprising; however, it highlights the challenges clinicians and researchers face in attempting to disseminate new approaches into existing care practices.

From the client’s perspective, self-reported willingness to use CBPT in their current treatment program was much higher than perhaps their clinicians anticipated (80% willing, but 34% exposed). This meant that 12 additional clients would have utilized SHADE, if offered, to assist them in managing their AOD use concerns. Clients willing to use SHADE tended to use cannabis at a higher level than their “unwilling” counterparts and reported more symptoms of stress on the DASS. Although a much larger study is required to properly test these trends, our results suggest that cannabis users and those feeling under stress might be more open to taking on SHADE. Previous research has indicated that, indeed, people using cannabis with comorbid depression do report superior improvements in cannabis use and depressive symptoms after receiving SHADE over face-to-face treatments [8] and that cannabis users in drug and alcohol treatment settings may be more suited to non face-to-face interventions [19].

Client self-reported depression, anxiety, stress, and alcohol and cannabis use declined over the study period. On average, alcohol and cannabis use, anxiety, and stress levels tended to be higher in clients who were exposed to the SHADE modules. It may be that severity of symptoms/AOD use served as the impetus for clinicians to use SHADE to augment their treatment with clients whose consumption of alcohol or cannabis was high or whose levels of anxiety or stress (but not depression) were high.

### Clinician openness to innovation, computer anxiety, and therapeutic alliance

#### Openness to innovation

There is little research to guide the description of innovativeness for mental health clinicians, crucial to the uptake of CBPT. While drug and alcohol clinicians are generally open to new and better treatments, most innovations find it difficult to make their way into clinical practice [20]. Generating new evidence-based treatments is not the most significant challenge for researchers; rather, it is increasingly seen that encouraging adoption and integration of evidence-based treatments into clinical practice is key [21]. Our study results indicate that while the clinicians in our study were above the norm on their readiness to accept and use technology and innovation in their clinical practice, this did not translate directly into the use of SHADE modules with the majority of their clients.

#### Computer anxiety

To our knowledge, no research has previously attempted to measure computer anxiety (or computer comfort) among AOD clinicians. There is, instead, a small body of research examining clinician perceptions and technological attitudes toward CBPT, albeit not in an AOD context [22]. As a group, participating clinicians in our study reported having relatively low-level computer anxiety. This would suggest that computer technology or the thought of using computer technology did not present as a limiting factor on a clinician’s decision to utilize SHADE during the study.

### Limitations and recommendations for future research

There are several limitations to the current study that are important to mention, not the least of which is the small sample size (clinicians and clients) and relatively small number of sessions available for examination. In addition, the data collection did not include an external check on client exposure to CBPT, and we are unable to verify the proportion of sessions for which clinician checklists were submitted versus all sessions conducted with clients. Instead, we used self-report data supplied by clinicians via a session-by-session checklist. Clinicians were asked to complete the brief clinician checklist for every client seen in every treatment session during the study period; but, for ethical reasons, we were unable to establish how compliant clinicians were with this request. Service data indicate that, on average, clients attend 4.5 treatment sessions per occasion of service with the CCAODS [23], suggesting that for clients engaged in our study (n = 77), we should have received approximately 347 clinician checklists over our 6-month recruitment period. Clinicians in our study submitted 304 clinician checklists, which is close to this estimate. As previously reported [23], we received 123 referrals to the project during the 6-month recruitment phase, resulting in 77 consenting clients for the larger study. Over a 2-year period, the CCAODS received 1,684 referrals to the service across 11 teams (including acute, nonacute, inpatient, and outpatient services). We targeted three teams from CCAODS for participation in the study. Based on these data, 459 referrals would have been received by these three teams over a 2-year period, or 115 over a 6-month period. We are therefore reasonably confident that clinicians referred most, if not all, clients they saw during the 6-month period for participation in the study.

Clinicians were invited by research staff to participate in the study, and all clinicians associated with our target service consented to participate. Consequently, the risk of selection bias may limit the generalizability of the findings. We also collected data from one treatment setting only that was staffed by relatively skilled and experienced clinicians. It is of note that we also only accessed clients who were already attending a drug and alcohol clinical service and therefore could not determine the unique contribution (if any) of CBPT over usual treatment. It remains unclear whether the results from this study would generalize across other drug and alcohol clinical services. Fully powered CBPT dissemination trials in different settings with different clinical groups are required. It would also be interesting to explore the use of CBPT among wait-list clients for drug and alcohol clinical services (i.e., clients who have sought treatment but have yet to attend their first treatment session). This was planned for the current study, but was unable to be implemented during the study period.

## Conclusion

In a small way, this study has contributed to our understanding of the factors that might influence a clinician’s decision to integrate CBPTs into their treatment plan. Clinicians were open to innovation, not anxious about using computers or technology, yet still only chose to utilize SHADE with 34 percent of clients. It has previously been suggested that eHealth in general, and CBPT in particular, is not only a *“technical development, but also a state-of-mind…an attitude, and a commitment…”* to using technology to improve health care [24]. The results of this study seem to support this notion, and point to the importance of committing significant support to adopt any new innovation, particularly CBPT.

## Competing interests

The authors declare that they have no competing interests.

## Authors’ contributions

FK-L, AS, and SC contributed to the study design and protocol development. FK-L and AS gained ethics approval for the trial, conducted the data analysis, and developed the initial draft of the manuscript. All authors contributed to manuscript preparation. All authors approved the final manuscript for submission.
